# The Pattern and Loci of Training-Induced Brain Changes in Healthy Older Adults Are Predicted by the Nature of the Intervention

**DOI:** 10.1371/journal.pone.0102710

**Published:** 2014-08-13

**Authors:** Sylvie Belleville, Samira Mellah, Chloé de Boysson, Jean-Francois Demonet, Bianca Bier

**Affiliations:** 1 Centre de recherche de l'Institut universitaire de gériatrie de Montréal (CRIUGM), Montréal, Québec, Canada; 2 Department of Psychology, University of Montreal, Montreal, Québec, Canada; 3 Leenaards Memory Centre at CHUV, Vaud University Hospital, Lausanne, Switzerland; University of California, San Francisco, United States of America

## Abstract

There is enormous interest in designing training methods for reducing cognitive decline in healthy older adults. Because it is impaired with aging, multitasking has often been targeted and has been shown to be malleable with appropriate training. Investigating the effects of cognitive training on functional brain activation might provide critical indication regarding the mechanisms that underlie those positive effects, as well as provide models for selecting appropriate training methods. The few studies that have looked at brain correlates of cognitive training indicate a variable pattern and location of brain changes - a result that might relate to differences in training formats. The goal of this study was to measure the neural substrates as a function of whether divided attentional training programs induced the use of alternative processes or whether it relied on repeated practice. Forty-eight older adults were randomly allocated to one of three training programs. In the SINGLE REPEATED training, participants practiced an alphanumeric equation and a visual detection task, each under focused attention. In the DIVIDED FIXED training, participants practiced combining verification and detection by divided attention, with equal attention allocated to both tasks. In the DIVIDED VARIABLE training, participants completed the task by divided attention, but were taught to vary the attentional priority allocated to each task. Brain activation was measured with fMRI pre- and post-training while completing each task individually and the two tasks combined. The three training programs resulted in markedly different brain changes. Practice on individual tasks in the SINGLE REPEATED training resulted in reduced brain activation whereas DIVIDED VARIABLE training resulted in a larger recruitment of the right superior and middle frontal gyrus, a region that has been involved in multitasking. The type of training is a critical factor in determining the pattern of brain activation.

## Introduction

Brain plasticity refers to the remarkable ability that cognitive systems have to modify their structure and functions in response to external or internal stimulation. There is ample evidence indicating that active processes of brain plasticity take place during early development, and following learning and sensory deprivation [1], [2]. Recently, there has been accumulating indications suggesting that brain plasticity is a lifelong phenomenon and that it also occurs in older age and throughout the course of age-related neurodegenerative disorders [3], [4], [5], [6], [7]. Recent studies have revealed that cognitive training can be a powerful means of directing behaviourally relevant reorganization in the adult brain, and brain imaging has been key in revealing processes of brain plasticity and compensation in older adults [8], [9]. Results of fMRI can reveal the patterns of brain changes that occur following training and can be used to indicate whether those programs increase the efficiency of specialized regions or whether they promote compensation through increased use of alternative processes. Furthermore, training-induced plasticity in older adults can be used as a model of brain reorganization and compensation in aging and provides information regarding the role of environmental stimulation on brain function over the lifespan.

However, important questions remain to be elucidated regarding the way cognitive training exerts its effect on brain changes, as different studies have revealed very different patterns of brain activation following cognitive training. One important question in our view is whether different training formats yield different patterns of activation. This is a critical component to better understand the effect of environmental stimulation and cognitive training on the brain. Training formats differ widely in terms of the cognitive component that they target (e.g., memory or attention) and in terms of the types of mechanisms that they engage or intend to impact upon (e.g., repeated practice to increase efficiency or learning new strategies to compensate via alternative pathways). Better understanding the type of brain changes induced by different training programs could help clinicians in selecting appropriate programs. For instance, clinicians may strategically select a program that improves a dysfunctional region (restoration), or one that allows the brain to support the impaired function by relying on unimpaired regions (compensation). Use of these strategies requires knowing which intervention has restorative effects and which has compensatory effects on the brain.

Understanding training-related brain changes is also relevant to theories of age-related compensation and plasticity. Older adults do not live in a vacuum, and they are constantly stimulated in their natural environment. Thus, a better understanding of the ways by which training shapes brain function might contribute to models of neural changes associated with environmental stimulation. In turn, theories of age-related compensation can guide predictions regarding the patterns of brain activation in older adults that should occur following training. Three of these models are particularly relevant here. The HAROLD model [3] suggests that the brains of healthy older adults compensate for the effect of aging by recruiting regions contralateral and homologous to the one typically involved in the task. The compensation-related utilization of neural circuits hypothesis (CRUNCH; [10]) also proposes that compensation is supported by increased activation in specialized brain regions, but that it can also occur by activating new, alternative regions. This would reflect strategic differences or a shift in the processes by which the task is completed. Both models predict that training should yield greater activation in regions not engaged by the task prior to training, and according to the HAROLD model, most likely, the contralateral homologue of the regions normally involved in the task. In contrast, the dedifferentiation model proposes that aging reduces the capacity to recruit specialized regions [11]. In this case, training should reduce rather than increase task-related brain activation (see Erickson et al. [12] for results in line with this prediction). Importantly, as none of these are training models, they do not address the effect that training formats can have on the patterns of compensation-related brain changes.

A few models have more specifically addressed the effects of cognitive training on the older adult brain and have included training format as an important factor to consider. Lovden and colleagues [13] proposed a theoretical framework to explain both age-related activation changes and training-induced brain changes in older adults. The model distinguishes different dimensions of the training format, one that modifies processing efficiency, and one that modifies the knowledge base or strategy registry. According to this framework, different types of training should have different effects on patterns of brain changes and on the extent and type of transfer expected to occur. Our own model, INTERACTIVE, suggests that training-induced activation changes depend on a number of interacting factors, including the format and characteristics of the training. It proposes that patterns of activation change are coherent with the underlying cognitive processes that could be modified by different types of training. For instance, the simple practice of a task should result in decreased activation within the brain regions involved in that task, due to more efficient processing in specialized regions. However, interventions involving teaching new strategies should result in increased activation of the alternative brain networks involved in learning those new strategies.

An analysis of the literature on training-induced brain changes in younger and older adults suggests that the training format is indeed a critical factor to explain differences in the patterns of brain changes. ***Decreased activation*** has been found in studies where younger adults repeated the task with no particular instruction or manipulation by the therapists or strong input-to-output mapping concordance (for a review see Chein & Schneider [14]). Similar findings were found in older adults when attention and working memory were trained with repeated practice and adaptive training [15]. The decreased activation was interpreted as resulting from a more efficient processing of the practiced task or a reduced reliance on controlled processing to accomplish the task. ***Increased activation or activation in new regions*** has also been reported in younger and older adults, and this has been suggested to reflect a change in the process used to complete the task - consistent with some of the predictions made by the CRUNCH model (Kelly, Foxe, & Garavan [16]). In older adults, the teaching of strategies or metacognitive training produced increased or new activation in brain regions that are expected to be engaged by the training; for example, the hippocampus for associative memory or the right parietal regions in the case of image-based mnemonics [17], [18], [19]. Intervention can also lead to a ***mixed pattern of increased and decreased*** recruitment. Braver, Paxton, Locke and Barch [20] found that following strategy training on task maintenance and updating, older adults showed a combination of increased activation in response to the cue and reduced activation in response to the probe, which was coherent with better selective attention. Erikson and collaborators [12] showed that practicing dual-task in healthy older adults reduced activation in the right ventrolateral prefrontal and dorsolateral prefrontal cortex, and increased left ventrolateral prefrontal activation. Belleville et al. [17] reported that mnemonic training in healthy older adults resulted in reduced activation during encoding; however, greater activation was found during retrieval.

Thus, studies of functional brain imaging following training in older adults found decreased activation, increased activation or a combination of increased and decreased activation. At first sight, this inconsistent pattern is troublesome, as it might suggest that functional brain imaging is not a reliable marker of brain training effects. However, these divergent results arise from a variety of different intervention modalities. The INTERACTIVE model suggests that training-induced activation changes depend on the characteristics of the training. Thus, predicting training effects requires an understanding of the mechanisms that are engaged or modified by the training program. Repeated practice appears to most often result in reduced activation, perhaps because it supports a more effective processing of the regions involved in the practiced task. In turn, training that involves teaching new strategies or that relies on metacognitive processes appears more likely to induce recruitment of new or alternative regions as proposed by the INTERACTIVE model.

Few studies have addressed the impact of training format in the interpretation of their findings, and no studies have compared activation changes as a function of the type of training. In this paper, we assess this interpretation directly and determine whether different patterns of brain activation in older adults can result from *repeated practice* or *strategic training*. The study measures the brain changes associated with three types of training, which are likely to differ in terms of mechanisms of action: (1) *repeated practice* of individual tasks in focused attention (SINGLE REPEATED), (2) *practice of divided attention* (DIVIDED FIXED), and (3) *training of strategic control of attention* (DIVIDED VARIABLE). Healthy older adults were randomized to one of the three training programs. Brain activation associated with performing individual tasks alone and both tasks combined was measured with fMRI prior to and following training. Our hypothesis is that decreased activation will be associated with repeated practice, whereas training that instantiates strategic control of attention strategies will be associated with new or increased activation in regions that are involved in controlled attention and multitasking. To our knowledge, this question has never been addressed empirically, and the training modalities have not been examined for the purpose of assessing compensation or training-induced activation changes.

## Materials and Methods

### 1. Ethics statement

This study was approved by the Institut universitaire de gériatrie de Montréal Human Ethics Committee and by The Regroupement Neuroimagerie/Québec (RNQ) committee. Informed written consent was obtained from all subjects according to the Declaration of Helsinki.

### 2. Participants

Forty-eight healthy, community-dwelling older adults were initially recruited to participate in this study through advertisements in seniors centres and magazines for seniors. Exclusion criteria included: alcoholism or substance abuse, head trauma, cerebral infection, epilepsy, cerebrovascular diseases, neurodegenerative disorders, mild cognitive impairment, major psychiatric illness, visual or motor limitations that would prevent their use of the computer, medication that could impact cognitive and cerebral functioning, and MRI incompatibility. Participants completed the Montreal Cognitive Assessment (MoCA; [21]), the Geriatric Depression Scale (GDS; [22]) to exclude persons with mild cognitive impairment or dementia, and the Coding and Similitude subtests of the WAIS-R [23] for characterization. They received a small financial compensation for their transportation expenses.

### 3. Design

**Figure 1:**
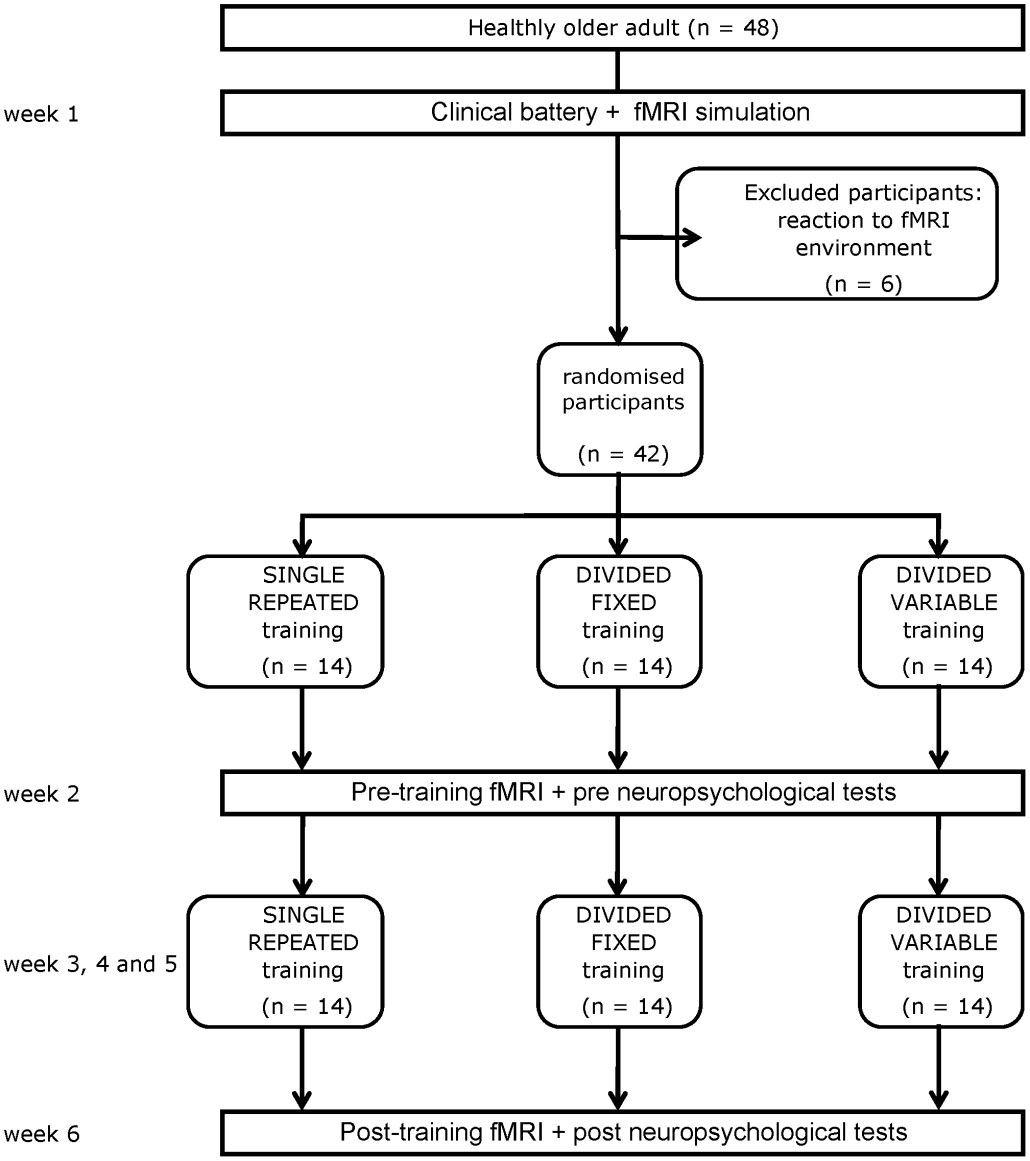
Flow chart according to the Consort reporting instructions.

Subject flow is shown in Figure 1 according to the CONSORT reporting instructions [24]. Participants were randomly assigned by an independent research assistant to one of the three training programs described below. There were two different versions of the pre- and post-session tasks, and their order was counterbalanced across participants.

### 4. Training method

**Figure 2:**
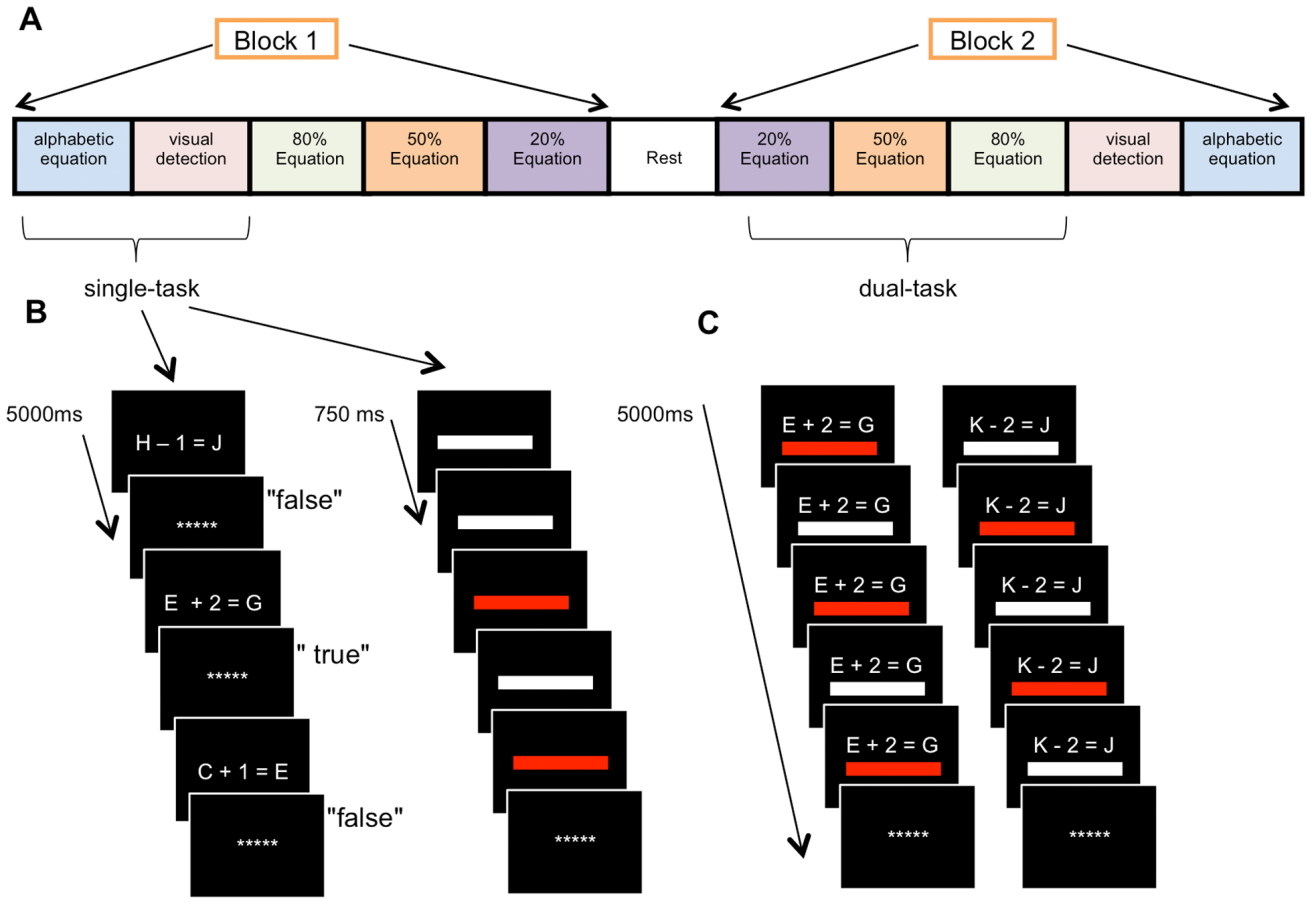
Experimental paradigm. Schematic representation of the task conditions order in fMRI (A), as well as the alphanumeric equation and visual detection tasks in both single-task (B) and dual-task (C).

Two tasks were used for the training: an alphanumeric equation task and a visual detection task. The tasks were either completed individually, in the condition of focused attention (single-tasking), or combined in the condition of divided attention (dual-tasking) (See Figure 2). Both tasks were run on Compaq Pentium d530 computers, and responses were given on the keyboard.

In the **alphanumeric equation task**, participants were asked to judge the accuracy of a set of visually presented alphanumeric equations. They were addition or subtraction equations constructed by combining a letter and a number (1 or 2) in the form: *letter* +/− *number*  =  *letter* (e.g., P+2 = R). The first letter was used as a starting point, and the number indicated the distance in terms of the number of intervening letters between the starting and end point. The sign indicated whether the end point was positioned before or after the starting point in the alphabet. In the example P+2 = R, the starting point is P and R is the letter that stands two positions ahead in the alphabet. This equation is thus correct. Training equations contained only letters that were part of the second half of the alphabet (from N to Z). Half of the equations were correct. Incorrect ones were formed by selecting a letter that was 1 or 2 positions away from the correct result. Equations were presented visually in the centre of a 17″ Viewsonic VE7106 Monitor, with white items over a black background. They were presented for a maximum of 3750 ms, with an interstimulus interval of 1500 ms filled with a centred fixation cross. Responses were made by pressing the “F” key with the left index finger when the equation was incorrect and the “J” key with the right index finger when the equation was correct.

In the **visual detection task**, participants were presented with series of white and red rectangles and were asked to detect the red one by pressing the space bar key with their left thumb. The rectangles were three inches high by thirty inches wide (1 cm by 8 cm) and were positioned just below the centre of the screen. They were presented for 500 ms with an interstimulus interval (ISI) of 250 ms. Forty percent of the rectangles were red, and they appeared in random order. In the divided attention conditions, five rectangles were presented while the equation was presented.

Training was provided in six one-hour sessions on weekdays over a period of two weeks, each separated by at least one day. Participants completed nine to thirteen blocks per session depending on the training program. The number of addition versus subtraction, one versus two steps, as well as correct versus incorrect equations were equivalent across blocks of trials. Accuracy and reaction time (RT) were recorded for both tasks. If a participant did not provide an answer within the maximum allotted time, the next equation was presented, and the trial was considered failed. To provide a baseline, all participants completed one block of each task under focused attention at the beginning and end of each session.

Participants received one of three training formats as described below.

#### Variable priority divided attention training (DIVIDED VARIABLE)

**Figure 3:**
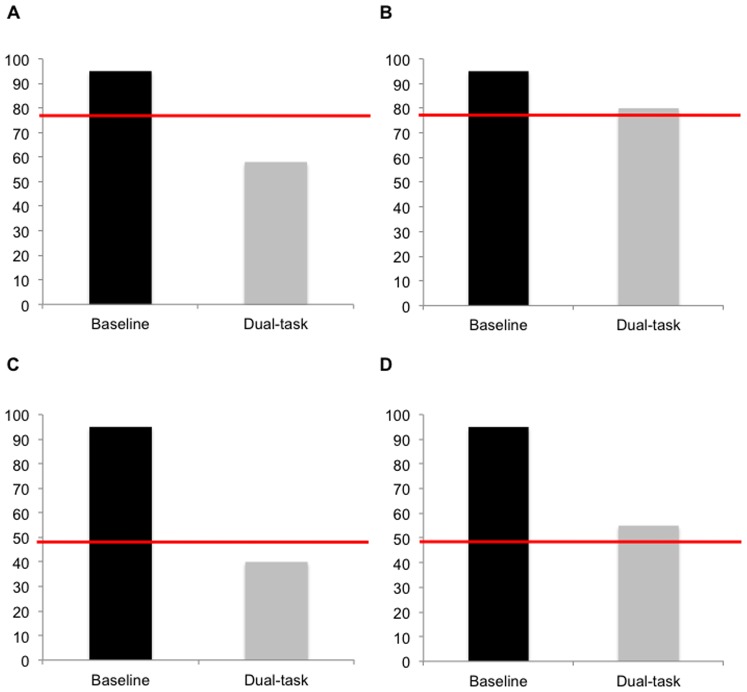
Feedback during variable training. Four examples of the histograms that provided visual feedback to the participants in the DIVIDED VARIABLE training group. The dark column represents the performance of alphabetical equation that was obtained under single-task baseline. The light column shows performance that was reached in the dual-task condition. The line represents the level of performance that was expected. (a; b) Examples of an 80% Equation trial where participants were asked to allocate 80% of their attention to the alphanumeric equation task: a) shows a trial where performance was below the expected threshold; (b) shows a trial where participants succeeded to obtain the expected level of performance. (c; d) Example of a 50% Equation trial where participants were asked to allocate 50% of their attention to the alphanumeric equation task: c) shows a trial where performance was below the expected threshold; (d) shows a trial where participants succeeded to obtain the expected level of performance.

Participants were asked to complete both tasks (alphanumeric equation and visual detection) simultaneously with divided attention. This was done under three conditions of attentional allocation: 80% Equation, 50% Equation, and 20% Equation. The 80% Equation condition required participants to allocate 80% of their attention to the alphanumeric equation task and 20% to the visual detection task (80/20), and vice-versa in the 20% Equation condition (20/80). In the 50% Equation condition, participants were asked to allocate an equal proportion of attention to both tasks (50/50). Each session comprised nine blocks of 20 trials of the dual-task (3 blocks per condition). Feedback was provided after each block. A histogram was presented to the participants, indicating their baseline level in focused attention, their performance on that trial, and the performance level that should have been attained according to the instructions. Participants were asked to draw their estimate of how they had performed, and their actual performance was then displayed with the histogram on the computer screen (see Figure 3). In this manner, participants were informed as to whether they had achieved the targeted allocation of attention, in order to better adjust their attention on the next block.

#### Fixed-priority divided-attention training (DIVIVED FIXED)

In the DIVIDED FIXED training program, participants were asked to complete the two tasks simultaneously with divided attention. They were instructed to allocate an equal amount of attention to each task. There was no feedback provided. Each session comprised nine blocks of 20 trials of the task.

#### Repeated single task training (SINGLE REPEATED)

In the SINGLE REPEATED training program, participants were asked to practice the alphanumeric equation task and the visual detection task individually under focused attention. Participants completed six blocks for one task and seven blocks for the other task in each session. The number of blocks was alternated across tasks, so that each task received the same amount of training overall. The order of tasks was alternated between sessions so that in a subsequent session, participants completed the opposite order. The starting task in session 1 was counterbalanced across participants. Thus, this training program was similar to the other two training programs except that the two tasks were not combined. Participants only received repeated practice on the individual tasks, and this was done under focused attention

### 5. fMRI methods

#### Task used in fMRI

Participants were scanned one week prior to and one week following training. The two tasks were completed separately in single-task and simultaneously in dual-task. The parameters of each task were identical to training except that the part of the alphabet used for the alphanumeric equation task was different (from A to M). The letter I was excluded so as to avoid confusion with the digit 1. The tasks were implemented using E-prime software (Psychology Software Tools, Inc.). Stimuli were presented using a projection system (Epson, EMP-8300), and they were visible to participants in a mirror attached to the head coil. Subjects' vision was corrected with goggles appropriate for MRI scanning as needed. Responses were given on fiber optic response pads (Brain Logics, BLBRS-FO-A) by pressing the appropriate button with the right index finger when the equation was incorrect, another button with the right middle finger when the equation was correct, and a third button with the right thumb when the red rectangle was shown.

Subjects performed the task in a blocked design with one run of five blocks. Each block was composed of a rest (20 sec) and the five task conditions (40 sec each): single-task alphanumeric equation, single-task visual detection, dual-task with equal division of attention (50% Equation: 50/50), dual-task with more emphasis on equation (80% Equation: 80/20), and dual-task with more emphasis on detection (20% Equation: 20/80). Each task condition comprised eight trials, and the order of presentation for the different conditions was varied within subjects. The single-task versus dual-task followed an ABBA order, and the emphasis conditions an ABCCBA order. The order of presentation for each condition is shown in Figure 2. No feedback was given. The task instructions were provided prior to each block and remained on-screen for 5 sec in the rest condition and 7.5 sec in the task condition. One week prior to the pre-training scanning, participants were trained on the fMRI procedure and practiced the task in a simulator that mimicked the fMRI environment (in terms of task, body position, sound, etc.).

#### Scanning parameters

Participants were scanned on a Siemens TIM Trio 3 T magnetic resonance imaging (MRI) system (Siemens Medical Solutions, Erlangen, Germany), using the Siemens 12-channel receive-only head coil at L'Unité de Neuroimagerie Fonctionnelle (UNF) du Centre de recherche de l'Institut universitaire de gériatrie de Montréal (http://www.unf-montreal.ca/siteweb/Home_en.html). Blood oxygenation level-dependent (BOLD) signal was acquired using a standard T2*-weighted gradient-echo EPI sequence (TR: 2500, TE: 30 ms, flip angle 90°, FOV 192×192 mm^2^, 38 slices, voxel size 3×3×3 mm^3^ with a gap of 0.6 mm-distance factor [20%], matrix size 64×64). Acquisition was in axial orientation co-planar with AC-PC, whole brain coverage. Order of acquisition was ascending. The functional images were acquired in one run, and the first three volumes were automatically discarded by the fMRI scanner. A structural image was acquired after the functional run using a high-resolution T1-weighted MPRAGE sequence (TR/TE 2300/2.98 ms, flip angle 9°, FOV: 256 mm 176 slices, voxel size 1 mm^3^, matrix size 256×256).

#### Image processing and analysis

Data were analyzed in MATLAB 7.1.2 (http://www.mathworks.com), using the statistical parametric mapping (SPM8) software (http://www.fil.ion.ucl.ac.uk/spm). The preprocessing consisted of the following steps: 1) motion correction, the temporal processed volumes of each subject were realigned to mean volume to remove the head motion, a mean realigned volume was created for each session, and the participants with more than 3 mm of translation in x, y, or z axis and 1° of rotation in each axis for one of the two sessions were removed; 2) slice-timing correction, the differences of each individual's slice acquisition times were corrected by slice timing to the middle volume, using SPM8 Fourier-phase shift interpolation; 3) co-registration of each subject's functional and anatomical data; 4) spatial normalization, the realigned volumes were spatially standardized into the Montreal Neurological Institute (MNI) space by normalizing with the EPI template via their corresponding mean image, and all the normalized images were resliced by 3 mm^3^ voxels; 5) smoothing, the normalized images were smoothed with a Gaussian kernel of 9 mm full width at half-maximum (FWHM). The two sessions (pre and post) were pre-processed separately.

The first level of statistical analysis carried out for each smoothed individual image was fixed effects analysis based on the general linear model (GLM) with a box-car response (HRF). GLM analysis was performed using regressors, generated by convolving the time course of the condition onsets and duration with canonical hemodynamic response function (HRF). There were six experimental conditions: rest (cross-fixation), single-task alphanumeric equation, single-task visual detection, dual-task 80% Equation (80/20), dual-task 50% Equation (50/50) and dual-task 20% Equation (20/80). The instruction before each condition was also modeled as a condition of no interest. Movement parameters estimated during realignment (translations in *x*, *y* and *z* directions, and rotations around *x*-, *y*- and *z*-axes) and a constant were also included in the matrix scanning run as variables of no interest. High-pass filter was implemented using a cut-off period of 256 s to remove the low-frequency drifts from the time series. Serial correlations in the functional MRI signal were estimated using an autoregressive (order 1) plus white noise model and a restricted maximum likelihood (ReML) algorithm. After estimating the parameters of the model, eight linear contrasts were calculated for each participant. Cerebral activation during single-tasking was calculated with the contrast *(single-task alphanumeric equation ≥ rest; single-task visual detection ≥ rest)*. Activation during dual-tasking was measured by separately calculating for each dual-task condition (80% Equation; 50% Equation; 20% Equation) with an interaction contrast: *(dual-task ≥ single-task alphanumeric equation) - (single-task visual detection ≥ rest).* This interaction contrast was used because it was shown to be the most appropriate method for comparing activity in the dual-task to that of the single-task [25], [26]. It allows for isolating the activation associated with dual-task, because it subtracts the activation associated with both tasks when performed individually.

The subject-specific contrast images were then further spatially smoothed (Gaussian kernel 6 mm full-width at half-maximum) and entered into a second-level random-effects analysis. First, activation associated with attention and attentional control was measured by pooling and analyzing the data from all participants during pre-training. One-sample *t*-tests were performed to measure the activation associated with dual-tasking in the different attention conditions, and paired t-tests were performed between dual-task 80% Equation (80/20) and dual-task 20% Equation (20/80) conditions to obtain effects of attentional control. We used paired t-tests to analyze the effects of training on activation under single-tasking comparing the contrasts *single-task alphanumeric equation ≥ rest* and *single-task visual detection ≥ rest* from pre- and post-sessions for each training group. To analyze the effects of training on activation during dual-tasking, we compared the contrast, *(dual-task* *≥ single-task alphanumeric equation) - (single-task visual detection task ≥ rest),* obtained in pre- and post-training of each condition *(80% Equation (80/20), 50% Equation (50/50) and 20% Equation (20/80)) and* for each training group. The resulting set of voxel values for each contrast constituted a map of the *t*-statistic [SPM(T)] that was thresholded at an uncorrected *P<*0.001 with 10 contiguous voxels. Again, this was done to isolate the changes in activation associated with the target conditions, relative to the changes in the baseline or single-task conditions.

Correlational analyses were used to assess whether the brain activation that occurred after training scans was associated with better performance on the behavioural tasks. The average beta values of the regions of interest (ROI) were extracted with MarsBaR [27] for each participant. The ROI is functionally defined as it corresponds to the regions that showed intervention effects in each condition. Performance on the critical dependent variables (RT on alphanumeric equation in single-tasking and dual-tasking cost) was then correlated with activation in brain regions found to be modified by the intervention. Pearson's correlations were performed using SPSS 19.0 (http://www.spss.com).

## Results

### 1. Clinical and cognitive results

Eight participants were excluded from the analysis; six experienced adverse reactions in the simulator and refused to continue with the fMRI examination, and two were excluded because of excessive head motion during the scan. The clinical and cognitive characteristics of the remaining 40 participants are shown in Table 1 as a function of the training program to which they were assigned. As shown in Table 1, the three groups were comparable in terms of demographic and clinical characteristics.

**Table 1:** Mean age, mean education, and mean score on clinical measures (S.D. in parentheses).

	SINGLE REPEATED Training (n = 12)	DIVIDED FIXED training (n = 14)	DIVIDED VARIABLE training (n = 14)	T value	P
Age	68.58 (8.16)	69.57 (5.81)	68.79 (5.13)	0.51	0.60
Education	14.17 (2.76)	15.21 (2.49)	16.00 (3.70)	1.32	0.28
Moca	28.25 (1.71)	27.43 (1.60)	27.29 (2.40)	0.58	0.57
GDS	1.63 (2.18)	1.43 (1.28)	2.21 (3.33)	0.87	0.43
Similarities WAIS-III subtest	12.25 (1.42)	12.14 (1.83)	12.36 (1.74)	0.06	0.94
Digit Symbol-Coding WAIS-III subtest	13.36 (1.36)	11.93 (1.59)	11.86 (1.99)	2.99	0.06

Table 2 presents behavioral performances in single-task alphanumeric equation and dual-task cost score (see below for computation of dual-task score), prior to and after training. As this paper focuses on brain activation, only summary analyses of behavioural data are presented here (see Bier et al. in press in Age, for more details).

**Table 2:** Performance in single-task alphabetic equation (reaction time and accuracy) and dual-task cost in pre and post sessions for each training group (S.D. in parentheses).

	Single-task performance				Dual-task cost+	
	Reaction time		Accuracy			
	Pre	Post	Pre	Post	Pre	Post
REPEATED training	2383 (121)	2307 (127)**	84.1 (3.1)	86.8 (3.1)**	0.45 (.04)	0.38 (.06)
DIVIDED FIXED training	2315 (105)	2146 (84)**	75.2 (4.6)	90.0 (3.6)**	0.53 (.05)	0.40 (.02)**
DIVIDED VARIABLE training	2466 (73)	2154 (100)**	77.3 (5.5)	89.8 (2.0)**	0.45 (.05)	0.39 (.04)*

+ Pooled across conditions.

^*^Task x Condition x Time Interaction, *p*<0.05.

^**^Main Time effect, *p*<0.01.

The training effect on performance when each task was completed under single-tasking was tested separately for each task (alphanumeric equation; visual detection) with Training program (SINGLE REPEATED, DIVIDED FIXED, DIVIDED VARIABLE) x Time (Pre, Post) ANOVAs using Reaction time (RT) and Accuracy (AC) as dependent variables. The analysis showed a main effect of Time for the alphanumeric equation task on both RT, *F*(1, 34) = 9.75, *p*<.001 (η^2^ = 0.22), 95% CI [63.99, 299.75] and AC, *F*(1, 34) = 14.8 (η^2^ = 0.30), *p*<.001, 95% CI [5.40, 17.52]. As shown in Table 2, all groups improved their alphanumeric performance when it was completed in single-task. No effect was found on the visual detection task.

To analyze the training effect on dual-tasking, a dual-task cost score was computed by combining the reaction time (RT) and accuracy (AC) for each task in the dual-task condition (DIVIDED) relative to the performance in single-tasking (SINGLE), with the following equation: {[(RT DIVIDED– RT SINGLE)/RT SINGLE] + [(AC SINGLE – AC DIVIDED)/AC SINGLE]}. In the equation, RT single and AC single represent performance in single-tasking for reaction time and accuracy. RT divided and AC divided represent performance in the dual-task conditions (80% Equation, 50% Equation or 20% Equation) for reaction time and accuracy.

This dual-task cost represents the proportional loss of performance in the dual-task condition as a function of performance in the single-task condition. A larger score represents a larger dual-task cost. A cost is determined separately for each task (i.e., alphanumeric equations vs. visual detection). This allows for examining the effect of attentional emphasis since the dual-task score should vary as a function of the way in which each task is prioritized. For instance, the dual-task score for the alphanumeric equations task should be lower when participants are instructed to emphasize the equations over the visual detection task (80% Equation) than when instructed to emphasize the visual detection over the equations task (20% Equation). Dual-task cost was used as a dependent variable in a repeated measure ANOVA with Time (pre, post), Priority instruction (80% Equation, 50% Equation and 20% Equation), and Task (alphanumeric equation; visual detection) as within-subject factors, and Training program (SINGLE REPEATED, DIVIDED FIXED, DIVIDED VARIABLE) as a between-subject factor. The analysis revealed a four-way interaction, *F*(1, 34) = 3.26, *p*<.01 (η^2^ = 0.16). To interpret the interaction, Time x Priority instruction x Task ANOVAs were done separately for each training program. The SINGLE REPEATED training resulted in no improvement with dual-tasking (no main effect of time or Time x Priority instruction x Task interaction (η^2^ = 0.00); *p* = .32 & *p* = .81, respectively). The DIVIDED FIXED training group showed a reduced overall dual-task cost from pre- to post training (main time effect, *F*(1, 34) = 6.97, *p*<.001 η^2^ = 0.45, 95% CI [0.04, 0.23]. Importantly, no Time x Priority instruction x Task (η^2^ = 0.027) was obtained indicating that participants did not improve their ability to vary their level of attention after training (*p* = .35). The DIVIDED VARIABLE training group showed a Time x Priority instruction x Task interaction, *F*(2, 33) = 5.17, *p*<.001 η^2^ = 0.34. After training, participants in that group showed a lower cost on the alphanumeric equation task when instructions required that the task was to be emphasized (80% Equation), and they showed a lower cost on the visual detection task when it was that task that was asked to be emphasized (20% Equation) (main effect of Priority instruction, *F*(2, 33) = 8.83, *p*<.001 η^2^ = 0.20), 95% CI [−0.31, −0.08]. This was not found prior to training. This indicates that after training participants in that group were able to modify their attentional priority in dual-tasking as a function of task instruction.

### 2. Brain activation related to attention (pre-training)

**Figure 4:**
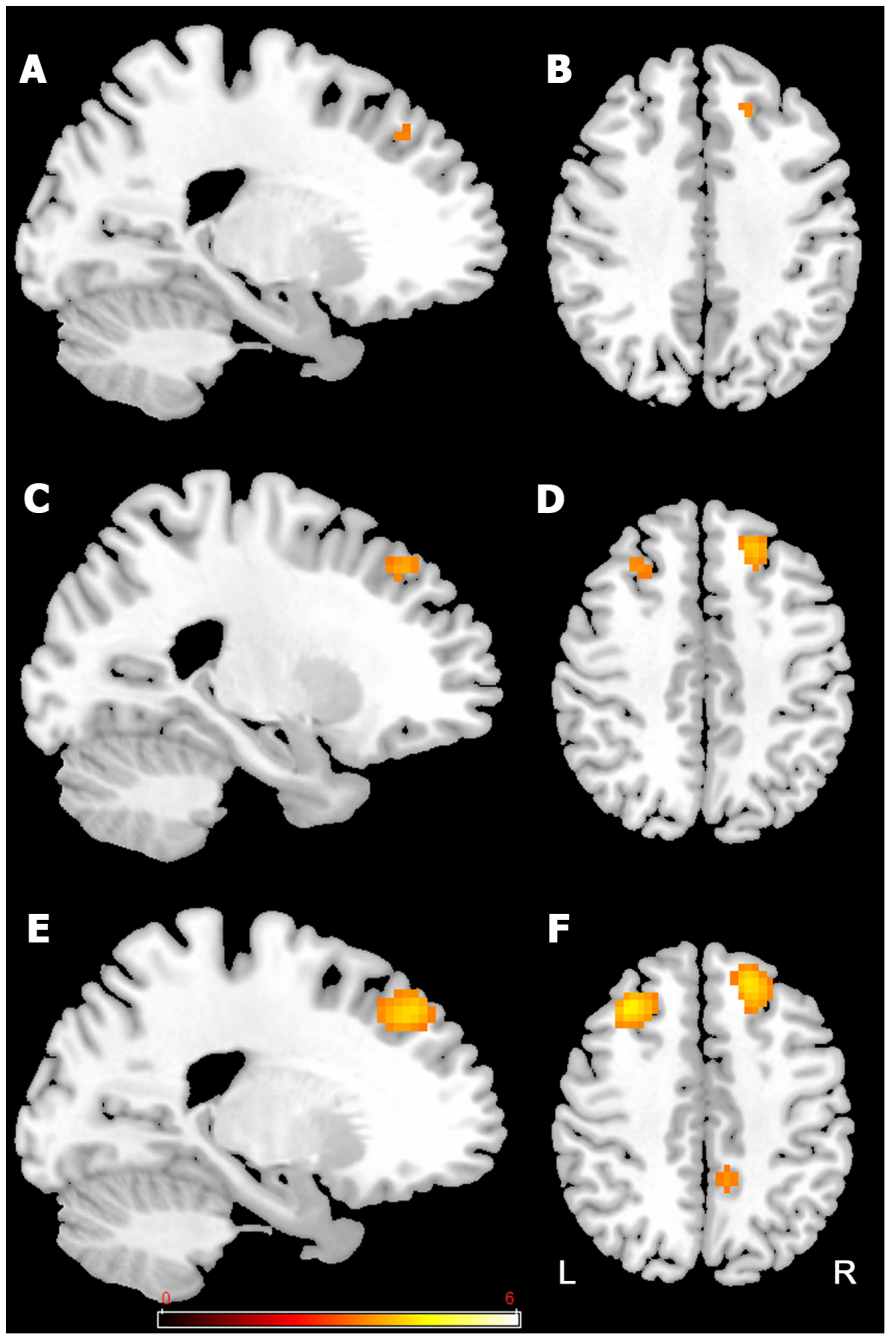
Activations related to dual-tasking prior to training (pre-training session). Network of prefrontal activation in dual-task, with more emphasis on equation (80% Equation (80/20)) in **A** and **B**; equal division of attention (50% Equation (50/50)) in **C** and **D**; and more emphasis on detection (20% Equation (20/80)) in **E** and **F.** The threshold for display is P<0.001, uncorrected, 10 voxels. Coloured bar is representative of t scores mentioned in Table 5. “L” denotes the left side of the brain, while “R” denotes the right side.

To determine whether training resulted in the activation of new brain areas or areas that were already activated prior to training, we first used the pre-training data from the entire group of participants to identify the areas of activation associated with dual-task (Figure 4 and Table 3). As shown in Figure 4 and Table 3, a network of prefrontal activation was recruited in the dual-task condition. The network was more active as persons moved their attentional priority from more attention on alphanumeric equations (80% Equation) to more attention on visual detection (20% Equation). Thus, activation related to *modulation of attention* was obtained by subtracting dual-task in the 80% Equation condition (80/20) from activation in the dual-task 20% Equation condition (20/80). Of note is the fact that the dual-task 50% Equation condition (50/50) showed an intermediate pattern of activation.

**Table 3:** Brain regions associated with dual-task at pre training by pooling all participants.

Activated areas (Brodmann area)	Cluster size	*x*	*y*	*z*	*t*-value
Dual-task 80% Equation (emphasis on equation)					
Right superior and middle frontal gyrus	15	21	32	38	3.67
Dual-task 50% Equation (equal division of attention)					
Right middle frontal gyrus (8,9)	37	24	32	41	3.97
Left middle frontal gyrus (8)	21	−27	26	47	3.64
Dual-task 20% Equation (emphasis on detection)					
Left middle frontal gyrus (8,9)	118	−30	23	44	4.56
Left superior frontal gyrus (9, 10)	71	−18	53	26	4.28
Right superior, medial and middle frontal gyrus (8,9,10)	223	21	32	38	4.27
left cerebelum	44	−18	−79	−43	3.81
Left anterior cingulate (24,32)	52	−6	32	8	3.78
Right precuneus and cingulate gyrus (7, 31)	35	15	−37	47	3.72
Right cerebellum	12	3	−49	−52	3.57

P<0.001 uncorrected, K = 10.

**Figure 5:**
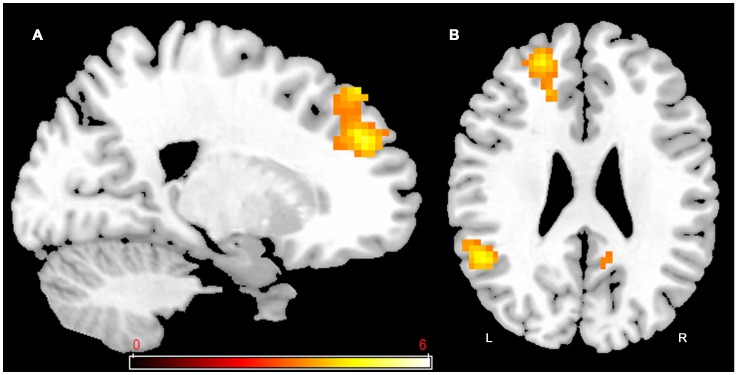
Activation-related to modulation of attention prior to training (pre-training session). Subtracting dual-task 80% Equation (80/20) from dual-task 20% Equation (20/80) involves activation in the left superior and medial frontal gyrus (A and B), and left superior temporal and left cingulate gyrus (B). The threshold for display is P<0.001, uncorrected, 10 voxels. Coloured bar is representative of t scores mentioned in table 5. “L” denotes the left side of the brain, while “R” denotes the right side.

During modulation of attention (Figure 5 and Table 4), clusters of activation were found in the left superior, medial frontal gyrus and anterior cingulate (areas 8-9-10), left inferior frontal gyrus (area 45), left cingulate (area 31), and left middle frontal gyrus (area 8). There was also activation in the left parietal and superior temporal gyrus (39-22) and left cerebellum. Many of these areas are typically involved in controlled attention [28].

**Table 4:** Brain regions associated with the modulation of attention at pre training by pooling all participants.

Activated areas (Brodmann area)	Cluster size	*x*	*y*	*z*	*t*-value
Left superior temporal gyrus (39,22)	162	−48	−55	17	5.65
Left cerebellum	44	−9	−49	−43	5.17
Left superior, medial frontal gyrus (8,9,10), anterior cingulate	235	−18	44	26	4.83
Left cingulate gyrus (31)	172	−3	−49	35	4.60
Left inferior frontal gyrus (45)	70	−51	26	8	3.92
Left middle frontal gyrus (8)	34	−42	14	50	3.79

(Dual-task 20% Equation > dual-task 80% Equation).

P<0.001 uncorrected, K = 10.

### 3. Brain activation related to training

The effect of training on brain activation was measured by comparing activation prior to training with activation after training and determining whether there was increased (Post>Pre) or decreased (Pre>Post) activation after training. This was done while participants performed each task under single-tasking and while they performed the two tasks in dual-tasking. The data is presented below for each training program. Contrast estimates (mean and standard deviation) for all training on significant contrasts are presented in Figure S1.

#### SINGLE REPEATED task training (SINGLE REPEATED)

**Figure 6:**
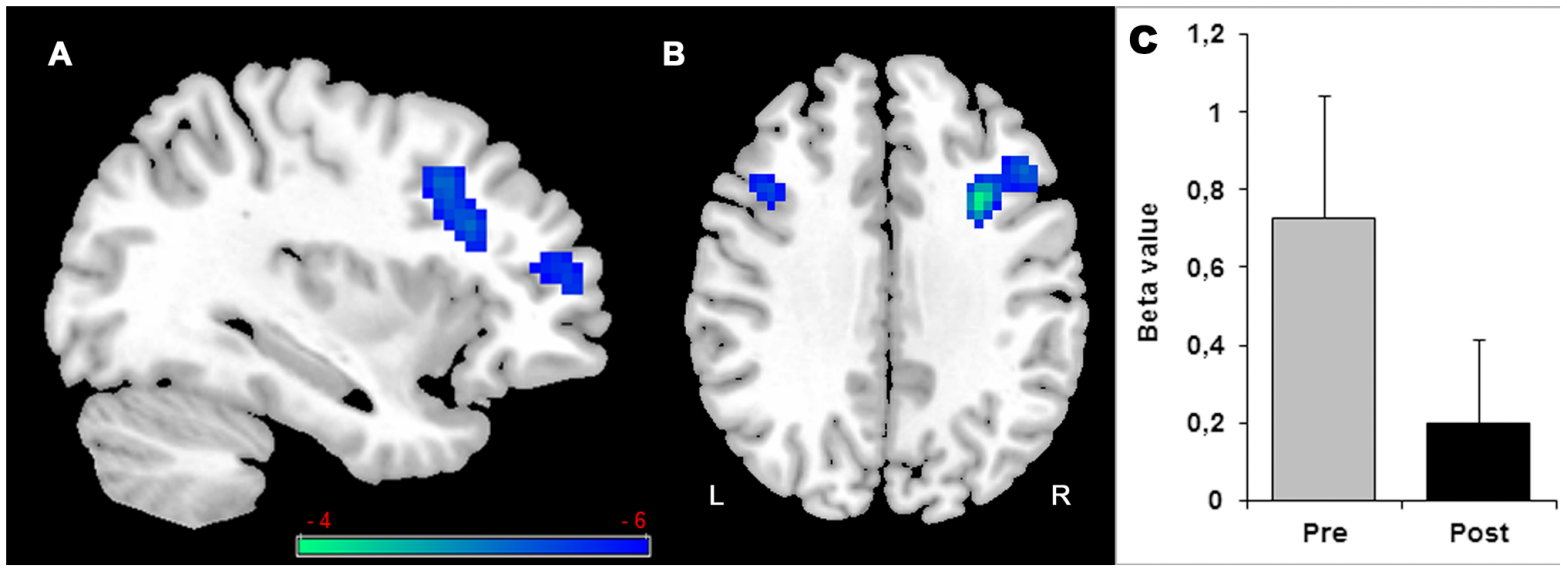
SINGLE REPEATED training effect. Decreased (Pre>Post) activation in single-task with alphanumeric equation is found in the right inferior and middle frontal gyrus (A and B) and left middle frontal (B). Histogram in (C) indicates the Beta value (activity estimates ± SE) in right inferior and middle frontal gyrus. The threshold for display is P<0.001, uncorrected, 10 voxels. Coloured bar is representative of t scores mentioned in Table 5. “L” denotes the left side of the brain, while “R” denotes the right side.

The group trained in the single training program (SINGLE REPEATED) showed areas of decreased post-training activation (Pre>Post) when completing the task under single-tasking, as shown in Figure 6 and Table 5. The paired t-test analyzing performance on the single-task alphanumeric equation indicated decreased post-training activation (Pre>Post) in the inferior and middle frontal gyri bilaterally and in the left thalamus (Figure 6). There was no post-training decrease in activation on either the visual detection task or when performing the two tasks under dual-task. In this group, there was no evidence for increased activation after training (Post>Pre) under single- and dual-task.

**Table 5:** Brain regions associated with training (Pre<Post or Post<Pre).

Activated areas (Brodmann area)	Cluster size	*x*	*y*	*z*	*t*-value
**SINGLE REPEATED training**					
Single-task alphabetical equation Pre>post					
Right inferior and middle frontal gyrus (46,9,10,45)	315	33	8	35	5.91
Left thalamus	96	−9	−31	8	5.37
Left middle frontal (9)	21	−36	14	32	4.57
**DIVIDED FIXED training**					
Single-task visual detection Pre>Post					
Rignt cerebellum	*11*	3	−79	−25	4.73
Right middle occipital gyrus (37,18,19)	*34*	33	−82	−1	4.68
Dual-task 50% Equation Post>Pre					
Left middle frontal gyrus (11,47)	13	−27	32	−16	4.52
Right superior and middle frontal gyrus (11)	16	27	35	−16	4.41
**DIVIDED** **VARIABLE training**					
Dual-task 80% Equation Post>Pre					
Right cerebellum	15	42	−76	−28	4.79
Dual-task 50% Equation Post>Pre					
Right superior and middle frontal gyrus (10)	12	27	56	23	4.78
Dual-task 20% Equation Post>Pre					
Right superior and middle frontal gyrus (10)	30	30	56	20	5.35

P<0.001 uncorrected, K = 10.

#### DIVIDED FIXED priority attentional training

Participants in the DIVIDED FIXED attention training program showed decreased post-training activation in the right cerebellum and right middle occipital gyrus (see Table 5), when completing the visual detection task under single-tasking (Pre>Post). No change was observed in post training activation on the single-task alphanumeric equation. When completing the dual-task 50% Equation (50/50), the group showed only small increases in post-training activation in the right and left middle frontal gyrus (area 11 and 47) (Table 5). Furthermore, there was no post-training change in activation when performing dual-task 80% Equation (80/20) or dual-task 20% Equation (20/80).

#### DIVIDED VARIABLE priority attentionnal training

**Figure 7:**
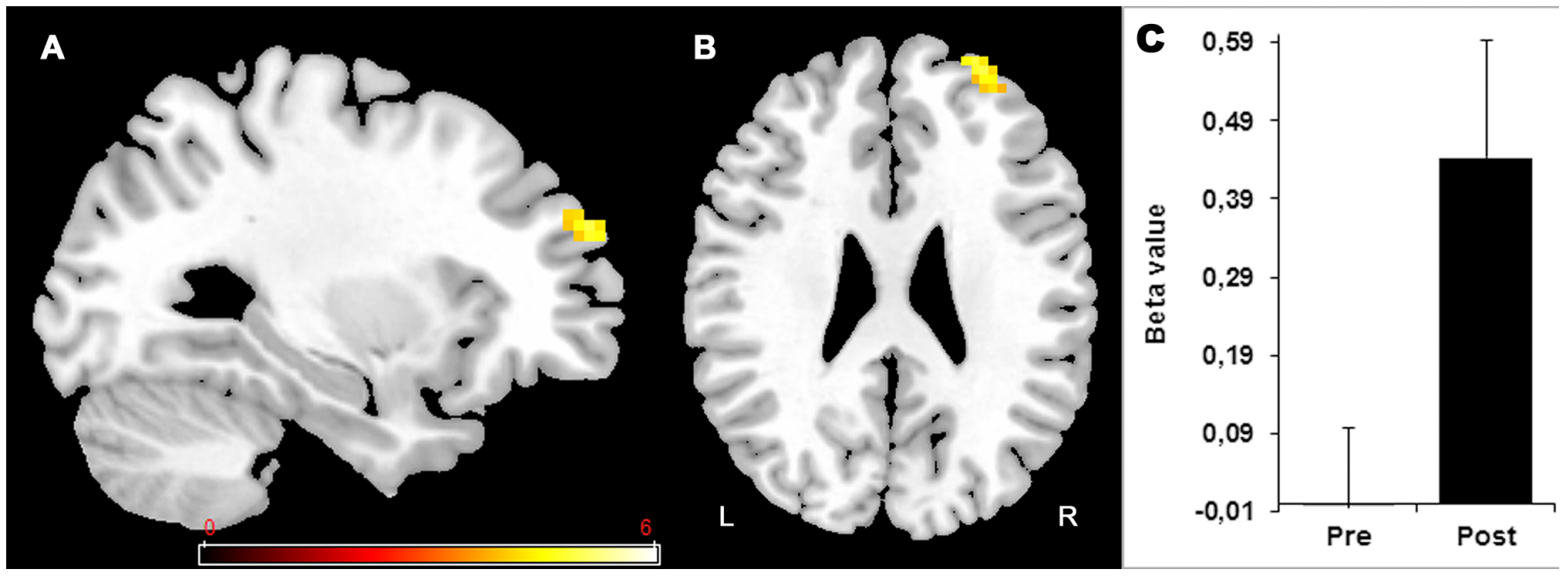
DIVIDED VARIABLE training effect. Increased (Post>Pre) activation in dual-task with more emphasis on detection (20% Equation (20/80)) is found in the right superior and middle frontal gyrus (10). Histogram in (C) indicates the Beta value (activity estimates ± SE) in the region showing increase activity in right superior and middle frontal gyrus during pre and post training session. The threshold for display is P<0.001, uncorrected, 10 voxels. Coloured bar is representative of t scores mentioned in Table 5. “L” denotes the left side of the brain, while “R” denotes the right side.

The group trained in DIVIDED VARIABLE attention showed neither reduced (Pre>Post) nor increased (Post>Pre) activation while completing the alphanumeric equation or visual detection tasks under single-tasking. While completing the dual-task, this group showed increased post-training activation (Post>Pre) in the prefrontal areas, as shown in Figure 7 and Table 5. The post-training (Post>Pre) in the 20% Equation dual-task condition was associated with a significant increase activation in the right middle frontal gyrus (area 10) (Figure 7 and Table 5). This group also showed increased post-training activation in the same right middle frontal gyrus (area 10) when completing the 50% Equation dual-task (Table 5). Finally, there was a small locus of increased post-training activation in the right cerebellum during the 80% Equation in dual-task (Table 5). In this group, completing the dual-task was not associated with reduced activation after training (Pre>Post).

**Figure 8:**
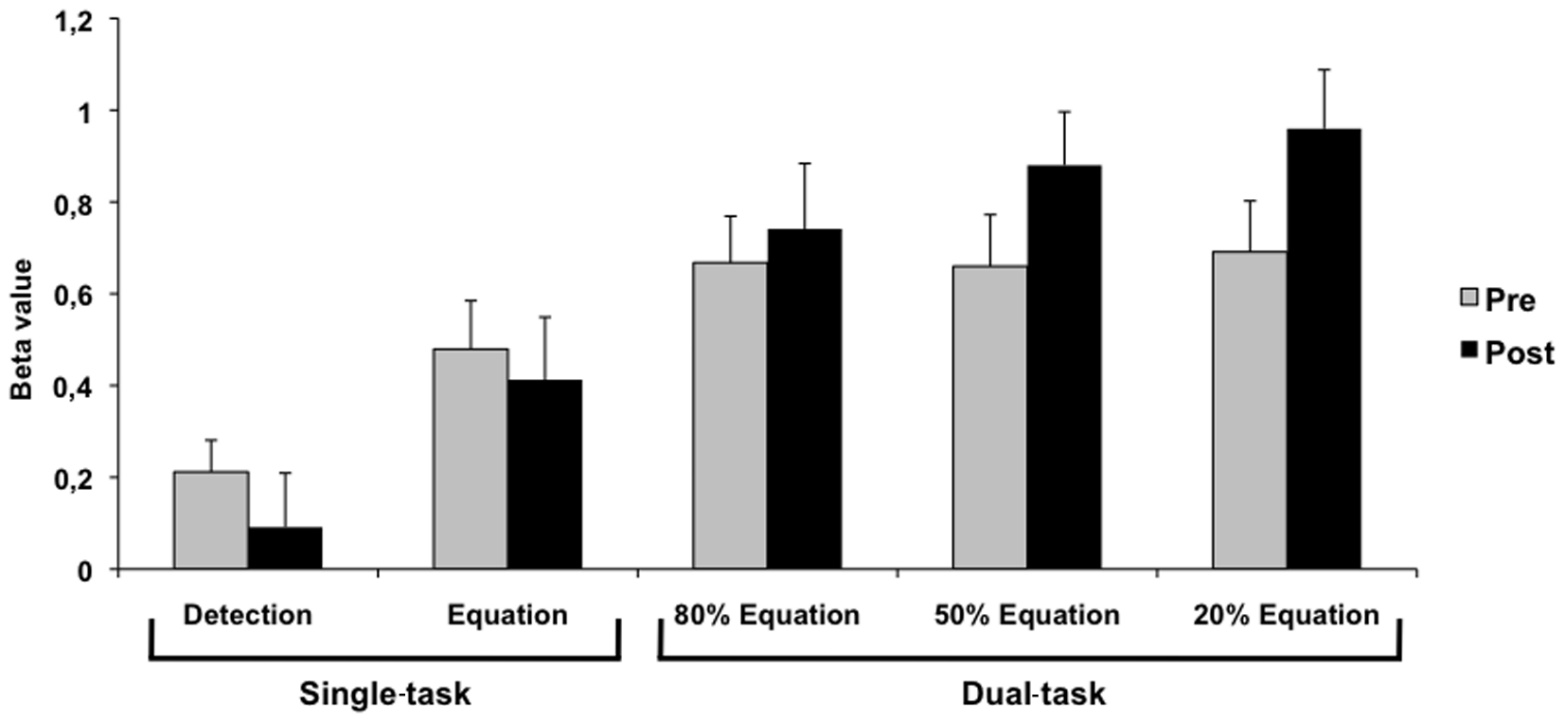
BOLD signal pre and post-intervention for the DIVIDED VARIABLE training. Beta value (activity estimates ± SE) in pre- and post-training sessions for single-task (visual detection > rest; alphanumeric equation > rest) and for each dual-task condition (80% Equation (80/20) > rest, 50% Equation (50/50) > rest and 20% Equation (20/80) > rest) in Right area 10 - the region showing post-training effect.

Figure 8 shows the BOLD signal found in the DIVIDED VARIABLE training in Right area 10 - the region showing the most consistent post-training effect - during the pre- and post-training sessions for single-task (visual detection > rest; alphanumeric equation > rest) and for each dual-task condition (80% Equation (80/20) > rest, 50% Equation (50/50) > rest and 20% Equation (20/80) > rest).

#### Correlation between performance and training-related activation

Table 6 shows the correlations for each group between post-training performance (RT in alphanumeric equation task) and activation in the right inferior and middle frontal gyrus (Brodmann's areas 46, 9, 10, 45), a region of interest for single-tasking, and between post-training attentional cost and activation in the right superior and middle frontal gyrus (Brodmann's area 10), a region of interest for the dual-task condition. Those regions were selected because they were changed by the training program in the group analysis. We were also interested in looking at Brodmans's area 10 as the literature had identified it as being specifically related to multitasking. Only participants in the SINGLE REPEATED training program showed a significant positive correlation between activation of the right inferior and middle frontal gyrus and performance in single-tasking, r = .56; *p*<.05. This positive correlation indicates that, at post-training, better performance in single-tasking (shorter RT) was associated with lesser brain activation in the right inferior and middle frontal gyrus. Similarly, only participants in the DIVIDED VARIABLE training program showed a significant correlation at post-training between attentional cost and activation of Brodmann area 10, r = −.55, p<.05; see Table 6. In this case, the negative correlation indicates that better post-training performance (i.e., lower dual-tasking cost) was associated with greater brain activation at post-training. Importantly, no correlation was found between dual-tasking cost and activation of this area prior to training (r = −.02, NS).

**Table 6:** Correlation between performance and post-training activation in (a) single- and (b) dual-task.

a) Training condition	Correlation value
SINGLE REPEATED training	0.56*
DIVIDED FIXED training	0.08
DIVIDED VARIABLE training	0.39

b) Training condition	Correlation value
SINGLE REPEATED training	−0.31
DIVIDED FIXED training	−0.26
DIVIDED VARIABLE training	−0.55*

**a)** Correlations between performance (reaction time (RT) on equations) and the beta value obtained in the right inferior and middle frontal gyrus during single-task (alphabetic equation) at post-training. The positive correlation indicates that smaller RTs are associated with less activation. **b)** Correlations between attentional cost and the beta value obtained in the right superior and middle frontal gyrus (area 10) during dual-task at post-training. The negative correlation indicates that smaller attentional costs are associated with more activation.

^*^significant at p<0.05.

## Discussion

In this study, we used fMRI to shed light on the brain processes involved in three different attentional training programs in healthy older adults. Overall, we found that the aging brain is highly plastic and that it responds in a coherent manner to different training methods. We also found that the type and loci of the brain response are largely dependent on the type of training provided as described below.

### Different training formats result in different behavioural and neural changes

Repeated practice on individual tasks (SINGLE REPEATED) made participants faster and more accurate when asked to solve the alphanumeric equations under single-tasking. In terms of neural changes when completing such a task, the SINGLE REPEATED training group showed reduced activation in the right inferior frontal gyrus, right middle frontal gyrus, left middle frontal gyrus, and in the left thalamus. Furthermore, correlations showed that better performance under single-tasking was associated with lesser activation of these regions at post-training, indicating that the effect found at the group level was coherent with that of the individual level. This suggests that the brain changes found here reflect successful compensation. Cabeza and Dennis [29] have indeed theorized that one empirical indication for successful compensation is a correlation between the brain changes and performance. In spite of their stronger performance on the individual alphanumeric equation task, the SINGLE REPEATED training group did not improve their ability to combine the equation task with the visual detection task in the dual-tasking condition. Thus, the dual-task cost was left unchanged by the training. Consistent with behavioural data, the SINGLE REPEATED training group showed no activation changes associated with dual-tasking.

Thus, and as hypothesized, training involving repeatedly practicing a task results in reduced brain activation. This is coherent with the INTERACTIVE model suggesting that as participants gain experience, there is a reduced need for activation because of increased efficacy of the recruited brain regions. In this case, the efficiency gained through experience did not result in a qualitative change in the way the task was completed or use of a different strategy because activation reduction is logically found in brain regions that were active prior to training. Similar findings were observed when younger adults practiced working memory tasks [14]. Erikson and collaborators [12] also reported post-training decreases in activation in the right prefrontal cortex - a region close to the one found here. It is interesting to note that areas showing decreased activation have been associated with monitoring processes of working memory. In particular, Stuss [30] and Stuss and collaborators [31] have proposed a model that relates different regions of the anterior frontal lobe to different executive functions. In this model, the dorsolateral cortex is involved in the online monitoring function of working memory. The alphanumeric equation task is a rather complex task that requires monitoring and updating the content of working memory. Our results suggest that enhancing capacity to solve the alphanumerical equations reduces the need for those controlled processes. It is remarkable that improvements following single-task training were only found on the alphanumeric equation task. This was found for all training groups; all of them improved on the equations task but not on the detection task. The detection task is very similar to a visual reaction time task and because it is an easy task, it mostly reflects processing speed. The literature shows that there is potential for processing speed to be increased in older adults. However, studies that have shown improved processing speed among older adults have typically used training programs that explicitly manipulate basic dimensions of the task; for instance, the time allowed for providing their response or the preparatory intervals (see Baron and Mattila [32]; Bherer and Belleville [33]). Lack of improvement on the detection task may be due to the fact that it was a very simple task, and that mere practice does not impact performance on such basic tasks. In turn, the alphanumeric equation task involve complex processes including working memory, monitoring, and updating, and these processes have been shown to be relatively plastic.

Repeated practice under divided attention in the DIVIDED FIXED attentional training program also resulted in a better ability to complete the alphanumeric equation task alone, as well as to divide attention between the alphanumeric equation and visual detection tasks when asked to combine the two. Thus, a lower dual-task cost was found post-training. In terms of brain changes, practicing under dual-tasking was followed by reduced activation when completing the visual detection task under single-tasking. It also resulted in small areas of increased activation in the middle frontal gyrus bilaterally during the 50% Equation (50/50) dual-task.

In the DIVIDED VARIABLE training group, participants were trained to variably control their attentional focus, to exert top-down control on the locus of their attention, and to improve their metacognitive abilities. We predicted that this training would increase their attentional control and lead to new or larger engagement of regions that are involved in multitasking and metacognition. As was the case for the other two training groups, participants in this group improved their ability to complete the alphanumeric equation task in the single-tasking condition. Critically, however, this training program was the only one to improve participants' ability to modulate their attentional priority according to task instructions. When participants were asked to prioritize the equations task, their dual-tasking score was reduced; the opposite was found when asked to prioritize the detection task. In terms of brain changes, the DIVIDED VARIABLE training resulted in increased activity in area 10 of the right prefrontal cortex, and this was found in two of the dual-tasking conditions (50% Equation and 20% Equation). None of these effects were found in the other two training programs. Importantly, we observed a correlation between greater activity in this region and better dual-tasking after DIVIDED VARIABLE training, indicating that the effect is coherent at the individual level. The correlation between increased brain activation and better dual-task performance also suggests that the activation changes found here reflect successful compensation [29].

Many studies in the attentional domain, have related area 10 to the coordination of multitasking [34], [35], [36], [37]. In line with this literature, the region was activated bilaterally under dual-tasking prior to training. In addition, Stuss [30] has proposed that this region is involved in orchestrating the basic executive functions needed to accomplish novelty tasks and is critical for metacognition. That it became more active following DIVIDED VARIABLE training indicates that older adults who were trained in this condition increased their reliance on brain regions associated with multitasking, perhaps because they engaged the coordinating processes necessary for completing such complex tasks. Interestingly, DiGirolamo and collaborators [38] have found that the brains of older adults recruit the medial frontal cortex even when not multi-tasking, perhaps as a way to compensate when task demands are important. Thus, this region might be an interesting component of compensatory processes in older adults.

### Models of Age-Related Brain Compensation

It is informative to relate patterns of brain loci modified by training to current theoretical frameworks of age-related brain compensation. One important question is whether activation results in reduced or increased activation. Another is whether training modifies activation in regions that were involved in the task prior to training (referred to here as specialized regions) or whether it modifies recruitment of regions that are not normally involved in the task (referred to here as alternative or latent regions [39], [40]).

Current models of compensation related to brain lesions or age indirectly address these issues. For instance, Pruvolic and collaborators [41] and Clement and Belleville [5] have proposed that compensation occurs naturally in the early course of age-related neurodegenerative diseases, and this is reflected in increased activation of the structurally impaired specialized regions that are typically involved in the task. In turn, the degeneracy model [42], [43] suggests that the complexity of brain interconnectivity makes different brain regions potentially apt at performing the same functions. Thus, loss of neurons in a specialized region might reveal latent systems in other regions that are either inhibited or left aside in non-impaired individuals. This latter view is coherent with current models of age-related compensation. The HAROLD model [3] proposes that the brains of older adults compensate by recruiting latent regions contralateral to those that are typically recruited by the task. The CRUNCH model [10] proposes that compensation is supported by increased activation of specialized brain regions and also by strategic recruitment of alternative regions. Thus, the degeneracy-type models predict that training should yield greater activation in alternative regions; that is, regions not engaged by the task prior to training. According to HAROLD, these would most likely be the contralateral homologues of the regions normally involved in the task.

The pattern of results we found is not easily reconciled with any of those models because the pattern varied widely as a function of the training format. DIVIDED VARIABLE training increased activation in right area 10, which is specialized for multitasking, and which was active bilaterally prior to training. At first sight, this pattern contrasts with the HAROLD model because this model suggests that the brains of older adults compensate by increasing activation in latent regions that are contralateral to those involved in the task. Similarly, the finding of reduced activation following repeated practice is not consistent with HAROLD, because the model suggests that compensation occurs through increased rather than decreased activation. Erikson and collaborators [12] have shown that attentional training reduces right prefrontal activation and increases left prefrontal activation in healthy older adults. This mixed pattern led to greater brain asymmetry post-training compared to pre-training, which is also contrary to the predictions of the HAROLD model. Interestingly, however, the reduced activity reported in the present study after repeated practice is found in the right hemisphere. It is possible that practice actually reduced the need to recruit from the contralateral region, which would then be consistent with HAROLD and the processing efficiency account proposed by the CRUNCH model.

### INTERACTIVE: a model of training-induced brain plasticity

Our major finding is that training-induced activation changes following attentional training differ strikingly as a function of training types. Importantly, these aforementioned models are concerned with naturally occurring compensation, and for this reason, they are not directly concerned by the effect of training. As a result, they have inherent limitations in accounting for findings related to differences of training-induced activation as a function of training types. In turn, the result is coherent with analyzing the type of cognitive processes that are engaged or modified by the training format. In this case, activation changes are considered to be not only biologically determined but also, determined by the cognitive mechanisms that are engaged or modified by training format. The training literature in aging shows results consistent with such an interpretation. For instance, Hampstead [44] reported increased activation in the hippocampus following training that increased associative memory capacities. Similarly, Belleville et al. [8], [17] and Nyberg et al. [19] reported increased activation in regions that are known to be involved in mental imagery and semantic elaboration, after training on imagery-based and elaborative encoding strategies. Braver, Paxton, Locke, and Barch [20] found that after strategy training on task maintenance and updating, older adults showed a combination of increased activation in response to the cue and reduced activation in response to the probe, which normalized their pattern of brain activity. In those cases, a task analysis of the training format and of the processes that it engages or changes would best determine the pattern and loci of brain changes following training.

INTERACTIVE is a training model that expends models of naturally-occuring compensation to interpret the data arising from training-induced activation changes. It suggests that activation changes depends on training modalities as well as on a complex interaction between those and the characteristics of the participants; for example, the type and extent of their brain changes, the availability of their cognitive reserve, and their level of expertise. In terms of training modalities, the INTERACTIVE model hypothesizes that repeated practice will result in decreased activation due to more efficient processing in specialized regions, whereas metacognitive training will result in activation of networks involved in controlled processing. In addition, the model distinguishes between training methods that promote compensation – by focusing on preserved functions- and training methods that promote restoration of impaired functions. Training protocols based on teaching mnemonics or promoting metacognition induce compensatory strategies and are more likely to result in increased activation. In contrast, restoration approaches focus on the impaired function and most often aim to increase the function by providing intensive and repeated practice and this might reduce activation in specialized regions. This might affect the choice of an appropriate training format, which may depend on whether the goal is to *increase* functioning in dysfunctional brain regions or to encourage *compensation* by making use of residual brain regions [45].

INTERACTIVE also identifies pre-training proficiency level as a factor in determining the pattern of change following training and this may interact with the type of training used. Metacognitive strategies could be taught to persons who already make use of those strategies. This might result in decreased activation as the training makes them more adept at using such strategies. For instance, we found that teaching mnemonics that promote deeper encoding reduced encoding-related activation in healthy older adults, but it increased encoding-related activation in persons with MCI [17]. We proposed that reduced activation occurred because older adults were attempting to use those strategies prior to training and they became more efficient at using them following training. Thus, activation changes not only depend on the type of training provided, but also on whether similar strategies were mastered prior to training and the level of mastery that was required. Clinical status is also an important parameter. Different clinical populations might be more sensitive to metacognitive, restoration, and compensation approaches. For instance, when the target region for restoration is too impaired at the structural level, one may wish to promote compensation processes that rely on unimpaired regions. In either cases, selection of the appropriate training program requires that the pattern of brain changes induced by a particular training format be well understood [9], [13].

### Limitations and remaining issues

We would like to recognize some of the limitations in this study and address remaining issues to explore. First, the sample size is relatively small when compared to typical randomized control trials. Note, however, that this sample size is in the upper range of those found for studies of training-induced brain changes in older adults [12], [15], [17], [18], [20], [44], [46], [47], [48]. Brain imaging studies are costly and demanding, and these constraints impose limitations on sample size, particularly for intervention studies where multiple scans are required and participants need to be split across different conditions. However, future studies will benefit from more powerful designs to broaden impact and increase generability of findings. More powerful designs will facilitate conducting studies that require a large number of participants (e.g., whole-brain connectivity), as well as investigating the impact of moderating variables on patterns of brain changes. With a larger sample size, we could have also used corrected p-values or direct group comparisons. Another important issue is that of generalization. One of the key concerns with training is showing transfer of benefits, not only to tasks very similar to the training itself, but also to untrained cognitive domains. It has been suggested that training basic cognitive processes, such as speed of processing or perceptual grouping, might result in greater transfer than training more complex processes. Measuring transfer was not the goal of this paper; however, knowing whether the neural changes found in the present study can support transfer to untrained cognitive tasks is an important question that should be addressed in future studies. Some of our findings can speak to this issue. Indeed, activation changes resulting from repeated practice are extremely different from those resulting from complex metacognitive training. However, the effect of repeated practice was found to be quite specific and limited to the task and condition that was trained, and results were not transferred to the dual-tasking condition.

## Summary

In summary, the attentional system of healthy older adults is highly plastic and behavioural and brain changes can be fostered by implementing relatively short training regimens. Importantly, however, the type of training appears to be a critical factor in determining the pattern of brain activation, as training formats vary in the effects they have on the brain. Practice reduces activation, perhaps through increased efficiency of the brain network implicated in the task for which expertise is developed. In turn, a training program that involves compensatory processes through the teaching of new metacognitive abilities is associated with increased activation. These findings can have a tremendous impact when selecting or designing training programs to prevent cognitive decline in older adults, as well as for those involved in rehabilitation of brain-damaged persons, because they provide a fine-grained analysis of the brain-related changes that can occur in response to different training formats. By showing that different programs can have dramatically different effects on cerebral activity, the results indicate that readaptation approaches should take into account not only the behavioural effects of particular intervention programs, but also their effects on the brain. Finally, these results and the model that we propose to account for them, can contribute to theories of brain compensation in aging and in age-related neurodegeneration.

## Supporting Information

Figure S1 **Contrast estimates (mean and standard error) for all training programs on significant contrasts (see in** **Table 5** **).**
(TIF)
