# Supplementary figures and images for: The Pattern and Loci of Training-Induced Brain Changes in Healthy Older Adults Are Predicted by the Nature of the Intervention
Source: PLoS One. 2014 Aug 13;9(8):e102710. doi: 10.1371/journal.pone.0102710 (PMC4131867; doi:10.1371/journal.pone.0102710)

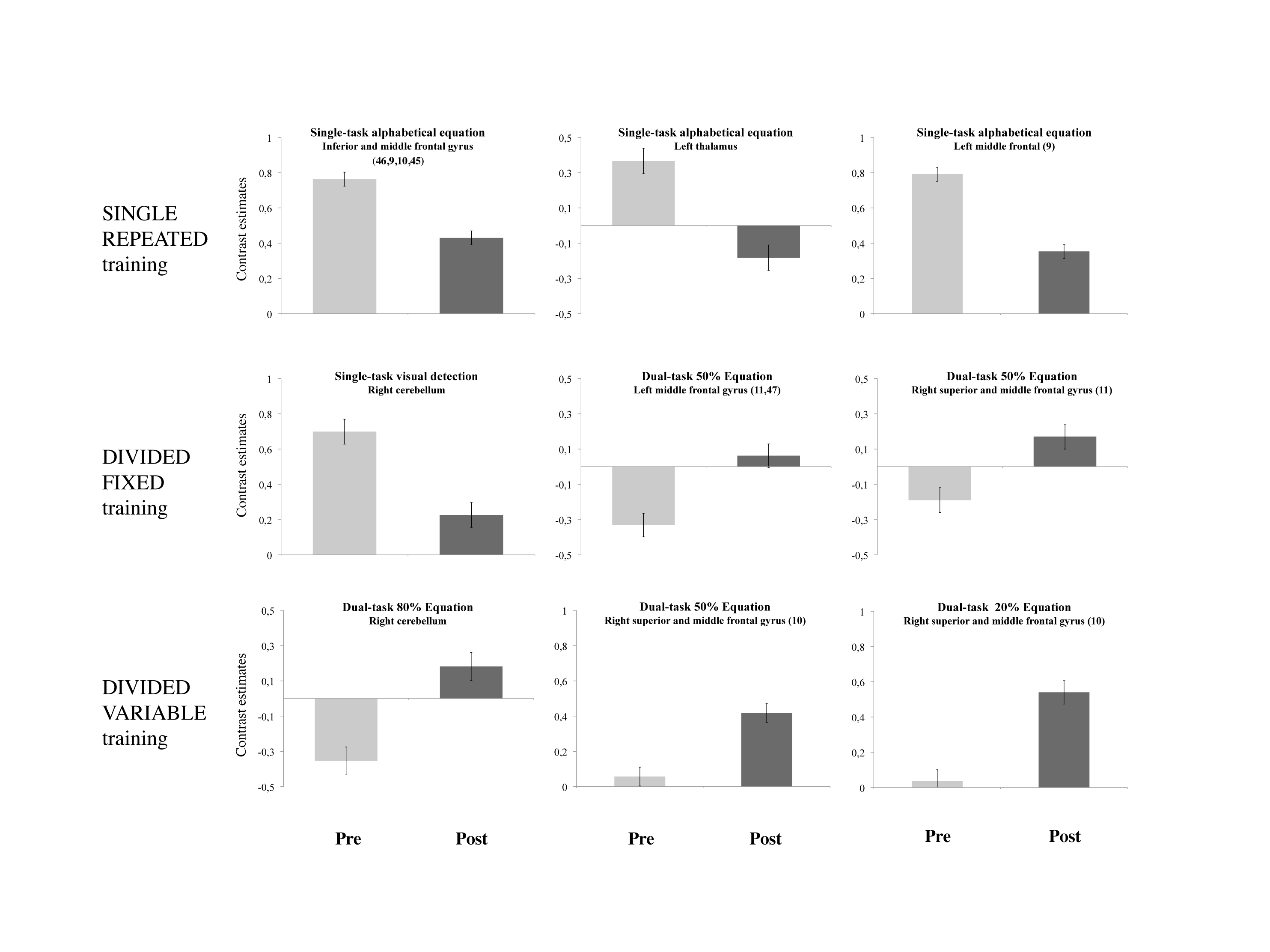

Supplement: Figure S1 — Contrast estimates (mean and standard error) for all training programs on significant contrasts (see in Table 5 ). (TIF) [file pone.0102710.s001.tif]
